# Drug response in organoids generated from frozen primary tumor tissues

**DOI:** 10.1038/srep18889

**Published:** 2016-01-07

**Authors:** Alex J. Walsh, Rebecca S. Cook, Melinda E. Sanders, Carlos L. Arteaga, Melissa C. Skala

**Affiliations:** 1 Department of Biomedical Engineering, Vanderbilt University, Station B, Box 1631, Nashville, Tennessee, 37235, USA; 2 Department of Cancer Biology, Vanderbilt University, 2220 Pierce Avenue, Nashville, Tennessee, USA; 3 Department of Medicine, Vanderbilt University, 2220 Pierce Avenue, Nashville, Tennessee, USA; 4 Breast Cancer Research Program, Vanderbilt- Ingram Cancer Center, Vanderbilt University.; 5 Department of Pathology, Microbiology & Immunology, Vanderbilt University Medical Center, 1301 Medical Center Drive 4918-A TVC Building, Nashville, Tennessee, 37232 USA

## Abstract

Primary tumor organoids grown in three-dimensional culture provide an excellent platform for studying tumor progression, invasion, and drug response. However, organoid generation protocols require fresh tumor tissue, which limits organoid research and clinical use. This study investigates cellular morphology, viability, and drug response of organoids derived from frozen tissues. The results demonstrate that viable organoids can be grown from flash-frozen and thawed tissue and from bulk tissues slowly frozen in DMSO supplemented media. While the freezing process affects the basal metabolic rate of the cells, the optical metabolic imaging index correlates between organoids derived from fresh and frozen tissue and can be used to detect drug response of organoids grown from frozen tissues. The slow, DMSO frozen tissue yielded organoids with more accurate drug response than the flash frozen tissues, and thus bulk tissue should be preserved for subsequent organoid generation by slow freezing in DMSO supplemented media.

Primary three-dimensional organoid culture of tumors is an attractive platform for studies of solid, epithelial tumors. Organoids contain all components of the original tissue, including malignant epithelial cells, endothelial cells, leukocytes, and fibroblasts. Three-dimensional organoid cultures recapitulate *in vivo* tissue structural organization, functional differentiation, chemical and mechanical signals, and therefore, may be more physiologically relevant than 2D cultures of primary or immortalized cells^1^,^2^,^3^. Traditionally, cancer models are limited to immortalized cell lines, xenografts established in mice, or genetically engineered mouse models. While these models are readily accessible and allow studies of cancer progression and drug testing, these models often do not represent human cancers and, as a result, do not consistently provide preclinical information of use in drug development^2^,^4^,^5^. Organoid culture of primary human tumors may overcome these limitations of traditional cancer models.

Organoid culture of primary tumor tissues enables dynamic studies of cancer development^3^, invasion^6^,^7^,^8^, and drug response^9^. Optical imaging, particularly multi-photon fluorescence imaging, is well suited to study organoids due to the spatial scale, depth of imaging, and functional fluorescence endpoints. Recently, we have shown that optical metabolic imaging (OMI) of organoids generated from primary breast tumors provides a dynamic and powerful assessment of drug response for both individualized patient treatment planning and exploratory studies of novel anti-cancer drugs^9^. OMI utilizes both the fluorescence intensity and lifetime of the metabolic co-enzymes, NAD(P)H and FAD, to detect early metabolic shifts in response to anti-cancer therapy. These metabolic shifts, detected non-invasively, correlate well with drug-induced inhibition of proliferation and/or induction of apoptosis within the organoids, as well as with *in vivo* drug response^9^. Due to its non-destructive nature and endogenous source of contrast, OMI is attractive for longitudinal studies of dynamic changes in cellular metabolism.

To date, all studies of primary tumor organoids have been performed on organoids generated from freshly harvested tumors^6^,^9^,^10^. However, fresh primary tumors are not always available. Further, tumor organoid-based research and patient treatment planning are limited to sites with nearby surgical rooms and research laboratories. Therefore, optimizing protocols for organoid generation using preserved tissues will allow research on banked tissues and eliminate the close proximity of biopsy source to laboratory constraint. Tissue is often preserved for biomedical research either as formalin-fixed paraffin embedded (FFPE) samples or flash frozen in liquid nitrogen. Preservation of cells in cell culture utilizes DMSO supplemented media and a slow-freezing procedure. This study investigates these latter two techniques for subsequent organoid generation.

This study tests the hypothesis that organoids can be grown from frozen/thawed tissues and that organoids generated by this approach will have the same response to drugs as organoids grown directly from fresh tissues. To test this hypothesis, organoids were generated from primary fresh tumor tissue and compared with organoids grown from primary tissues frozen in two ways: (1) flash frozen, or (2) slowly frozen in tissue culture media + 5% DMSO. Organoid viability was assessed by immunofluorescence (IF) assessment of proliferation and apoptosis proteins, Ki67 and cleaved caspase 3, respectively. Organoid drug response was assessed with OMI and IF. Both freezing protocols were performed on two xenograft models of HER2-overexpressing breast cancers, BT474 and HR6 tumors, to compare the two freezing techniques for optimal organoid viability and drug response. The flash-frozen experiments were additionally performed on two primary human breast cancer biopsies to evaluate the use of frozen tissues maintained in tissue banks.

## Results

### Organoids Derived from Frozen-thawed Xenograft Samples Retain Viability

**Figure 1:**
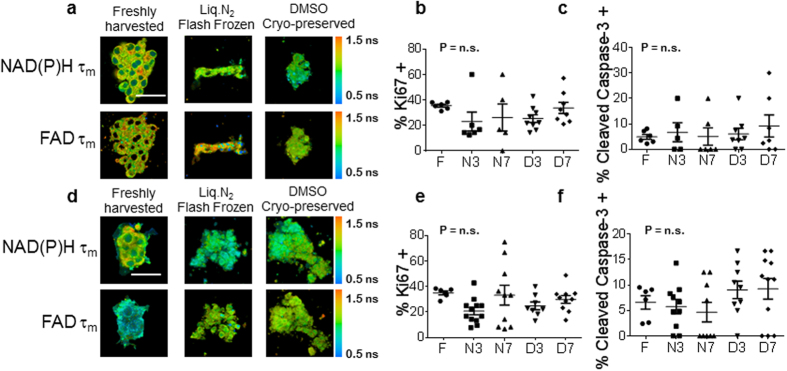
Organoids derived from frozen-thawed xenograft tumors are viable. (**a**) Representative NAD(P)H τ_m_ and FAD τ_m_ images of BT474 organoids derived from fresh, flash-frozen, and DMSO cyro-preserved tissues. Scale bar is 50 μm. (**b**) Ki67 and (**c**) cleaved caspase 3 staining of control organoids derived from fresh, flash-frozen and DMSO frozen BT474 tumors. (**d**) Representative NAD(P)H τ_m_ and FAD τ_m_ images of HR6 organoids derived from fresh, flash-frozen, and DMSO cyro-preserved tissues. Scale bar is 50 μm. (**e**) Ki67 and (**f**) cleaved caspase 3 staining of control organoids derived from fresh, flash-frozen and DMSO frozen HR6 tumors. F = Fresh, N3/7 = Flash frozen and grown for 3/7 days, D3/7 = DMSO frozen and grown for 3/7 days. n = 5–12 organoids.

Organoids were successfully grown from fresh, flash-frozen, and DMSO frozen samples of BT474 xenograft tumors (Fig. 1a, Supplementary Fig. 1). The organoids generated from flash-frozen and DMSO frozen BT474 tissue samples had similar expression of Ki67 and cleaved caspase 3 as compared to organoids derived from the fresh BT474 tumor sample (Fig. 1b,c). Likewise, the organoids generated from flash-frozen and DMSO frozen HR6 tumor samples grew (Fig. 1d, Supplementary Fig. 1) and had similar percentages of Ki67 and cleaved caspase 3 positively stained cells as compared to organoids generated from fresh HR6 tissue (Fig. 1e,f).

### Morphology of Organoids Derived from Frozen-thawed Xenograft Samples is Different for BT474 Cells and Retained for HR6 Cells

**Figure 2:**
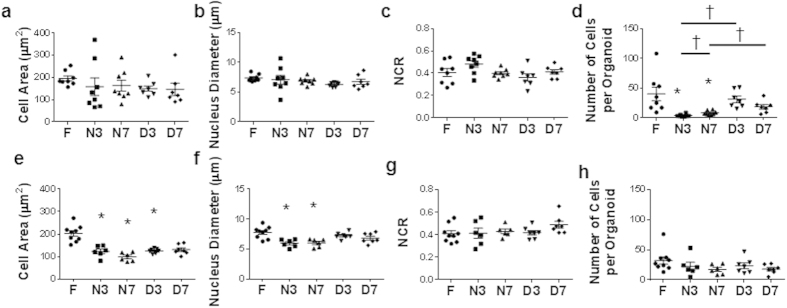
Morphology of organoids derived from frozen-thawed xenograft tumors may differ from organoids derived from fresh tissue. (**a**) Cell area, (**b**) nucleus diameter, and (**c**) nucleus to cytoplasm ratio (NRC) for BT474 organoids derived from fresh and frozen/thawed tissues. (**d**) Number of cells per organoid for BT474 organoids derived from fresh and frozen/thawed tissues. (**e**) Cell area, (**f**) nucleus diameter, and (**g**) nucleus to cytoplasm ratio for HR6 organoids derived from fresh and frozen/thawed tissues. (**h**) Number of cells per organoid for HR6 organoids derived from fresh and frozen/thawed tissues. F = Fresh, N3/7 = Flash frozen and grown for 3/7 days, D3/7 = DMSO frozen and grown for 3/7 days. *p < 0.05 versus Fresh. †p < 0.05 as indicated. n = 6–9 organoids.

Next, cellular morphology was quantified and compared between organoids generated from fresh and frozen tissues. No change in cell area, nucleus diameter, or nucleus to cytoplasm ratio (NCR) was detected for the cells within organoids generated from frozen-thawed BT474 tumor samples compared to organoids generated from fresh BT474 tumor samples (Fig. 2a–c). However, there was a significant reduction in the number of cells within each organoid for the organoids generated from flash-frozen BT474 tumors compared to organoids generated from fresh tissues, for organoids grown for both 3 and 7 days (Fig. 2d). A significant reduction in number of cells within each organoid was not observed for organoids generated from BT474 DMSO frozen tissues (Fig. 2d).

In contrast to the BT474 results, the cells grown from frozen HR6 tumor samples showed morphological differences compared to cells grown from fresh tumor samples. The cell area of HR6 cells was significantly reduced for cells within organoids generated from flash-frozen sections grown for 3 or 7 days, and in organoids generated from DMSO frozen tissue grown for 3 days (Fig. 2e). Likewise, the nucleus diameter was significantly reduced for cells within organoids generated from flash-frozen tissue (Fig. 2f). The NCR of HR6 cells did not significantly change due to either freezing method (Fig. 2g). Also, no significant change in the number of HR6 cells within each organoid was detected for either freezing protocol or length of growing time (Fig. 2h).

### Drug Response of BT474 Organoids Generated from Frozen Tissues Matches Drug Response of Organoids Generated from Fresh Tissues

**Figure 3:**
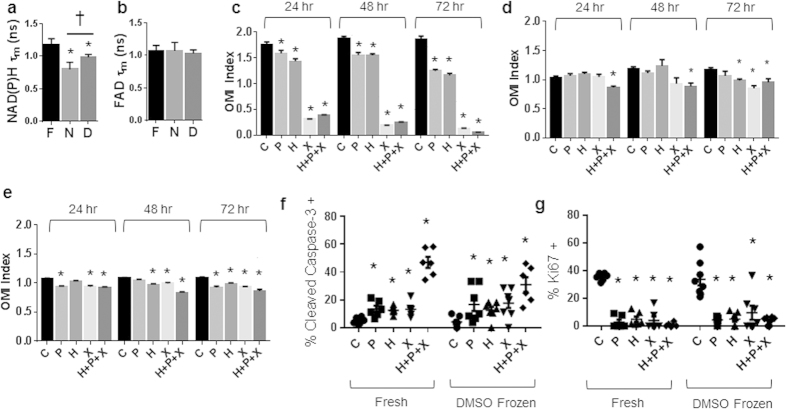
Drug response of BT474 organoids derived from frozen-thawed tumors matches drug response of organoids derived from fresh tissue. (**a**) NAD(P)H τ_m_ and (**b**) FAD τ_m_ measured from BT474 organoids derived from fresh or frozen tissues grown for 7 days. *p < 0.05 vs Fresh, †p < 0.05, as indicated; mean+/− SE. n = 30–300 cells. F = Fresh, N = Flash frozen, D = DMSO frozen. Drug response of BT474 organoids derived from fresh tissue (**c**), flash-frozen tissue grown for 7 days (**d**), and DMSO frozen tissue grown for 7 days (**e**) at 24, 48, and 72 hr. *p < 0.05 versus control; mean +/− SE. n = 30–300 cells. Cleaved caspase 3 (**f**) and Ki67 (**g**) staining of 72 hr drug-treated organoids derived from fresh and DMSO frozen (grown for 7 days) tissues. *p < 0.05 versus control, within Fresh or DMSO Frozen. n = 5–8 organoids. C = Control, P = Paclitaxel, H = Trastuzumab, X = XL147.

Optical metabolic imaging (OMI) endpoints were evaluated for metabolism differences between organoids grown from fresh tissues and organoids grown from frozen/thawed tissues. For the BT474 tumors, NAD(P)H τ_m_ was significantly decreased in cells from organoids generated with both freezing methods on day 7 (Fig. 3a). However, FAD τ_m_ showed no significant differences between organoid generated from fresh and frozen BT474 tumors (Fig. 3b).

The 72-hour time course for drug response of BT474 organoids generated from fresh tissues showed a significant reduction in the OMI index with paclitaxel (P), trastuzumab (H), XL147 (X), and the combination, H + P + X at 24 hr, 48 hr, and 72 hr of drug treatment (Fig. 3c). For the BT474 organoids derived from flash-frozen tissues grown for 7 days prior to drug treatment, the OMI index was significantly reduced with only the combination treatment, H + P + X, at 24 and 48 hr of drug exposure (Fig. 3d). By 72 hr, trastuzumab, XL147, and H + P + X induced significant reductions in the OMI index (Fig. 3d). Organoids derived from the BT474 tumor sample slowly frozen in DMSO supplemented media, showed response to anti-cancer drugs (Fig. 3e). By 72 hr, paclitaxel, trastuzumab, XL147, and H + P + X treated organoids all had significant reductions in OMI index (Fig. 3e). Immunofluorescence staining of Ki67 and cleaved caspase 3 of organoids derived from fresh and DMSO frozen BT474 tumors treated for 72 hr with anti-cancer drugs, revealed increased cleaved caspase 3 expression in organoids treated with paclitaxel, trastuzumab, XL147, and H + P + X (Fig. 3f). Likewise, reduced expression of Ki67 was observed in organoids treated with all anti-cancer therapies (Fig. 3g).

### Drug Response of HR6 Organoids Generated from Frozen Tissues Matches Drug Response of Organoids Generated from Fresh Tissues

**Figure 4:**
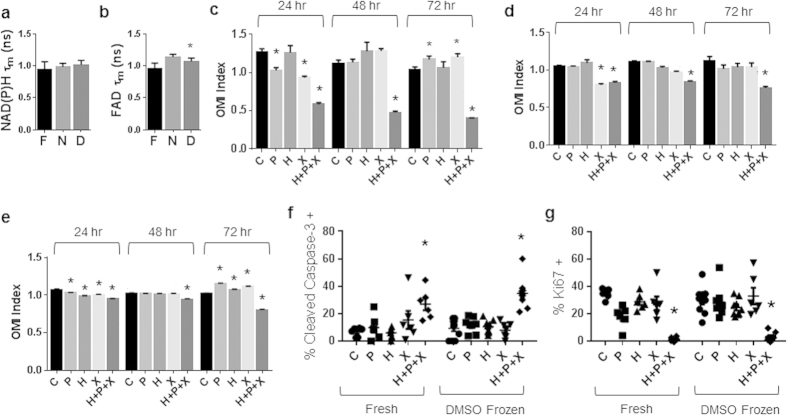
Drug response of HR6 organoids derived from frozen-thawed tumors matches drug response of organoids derived from fresh tissue. (**a**) NAD(P)H τ_m_ and (**b**) FAD τ_m_ measured from HR6 organoids derived from fresh or frozen tissues grown for 7 days. *p < 0.05 vs. Fresh; mean +/− SE. F = Fresh, N = flash frozen, D = DMSO frozen. n = 30–300 cells. Drug response of HR6 organoids derived from fresh tissue (**c**), flash-frozen tissue grown for 7 days (**d**), and DMSO-frozen tissue grown for 7 days (**e**) at 24, 48, and 72 hr. *p < 0.05 versus control; mean +/− SE. n = 30–300 cells. Cleaved caspase 3 (**f**) and Ki67 (**g**) staining of 72 hr drug-treated organoids derived from fresh and DMSO frozen (grown for 7 days) tissues. *p < 0.05 versus control, within Fresh or DMSO Frozen. n = 6–11 organoids. C = Control, P = Paclitaxel, H = Trastuzumab, X = XL147.

The effect of freezing HR6 tumors before generating organoids was also evaluated with OMI of organoid drug response. No change in NAD(P)H τ_m_ was observed between organoids generated from fresh HR6 tumors and flash frozen or DMSO frozen tumors on day 7 after organoid generation (Fig. 4a). A slight increase in FAD τ_m_ was observed in organoids generated from flash-frozen HR6 tumors compared to organoids generated from fresh HR6 tumors (p < 0.05, Fig. 4b); however, no significant change in FAD τ_m_ was observed in the organoids generated from DMSO frozen HR6 tissue (p > 0.05, Fig. 4b).

Organoids generated from fresh HR6 tumors have a significantly reduced OMI index at 24 hr due to paclitaxel, XL-147, and H + P + X treatment (Fig. 4c). By 48 and 72 hr, only H + P + X induces a significant reduction in the OMI index (Fig. 4c). For the HR6 organoids derived from flash-frozen HR6 tumors, at 24 hr, both XL147 and H + P + X induce significant reductions in the OMI index (Fig. 4d). By 48 and 72 hr, only the H + P + X treated organoids have a significant reduction in OMI index (Fig. 4d). Likewise, HR6 organoids derived from the DMSO frozen tumor showed significant reductions in OMI index with paclitaxel, trastuzumab, XL147, and H + P + X treatment at 24 hr (Fig. 4e). By 48 hr and 72 hr, only the H + P + X treatment induced a significant reduction in the OMI index (Fig. 4e). At 72 hr, the paclitaxel and XL147 treated HR6 organoids induced a significant increase (p < 0.05) in the OMI index in both organoids generated from fresh (Fig. 4c) and DMSO frozen tumors (Fig. 4e), but this increase was not detected in the organoids derived from flash-frozen tumors (Fig. 4d). Immunofluorescence staining of cleaved caspase 3 and Ki67 of organoids derived from fresh and DMSO frozen tumors treated for 72 hr revealed an increased proportion of cells stained cleaved caspase 3 positive and a reduced proportion of cells stained Ki67 positive in H + P + X treated HR6 organoids (Fig. 4f,g).

### Organoids Derived from Frozen-thawed Human Breast Cancer Biopsies Retain Viability

**Figure 5:**
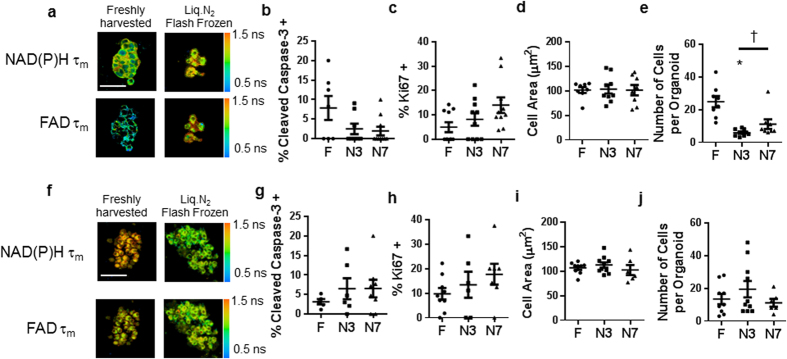
Organoids derived from frozen-thawed human tumors are viable. (**a**) Representative NAD(P)H τ_m_ and FAD τ_m_ images of organoids derived from fresh and flash frozen/thawed Patient Sample 1. Scale bar is 50 μm. (**b**) Cleaved caspase 3 staining, (**c**) Ki67 staining, (**d**) cell area, and (**e**) number of cells per organoid for organoids derived from fresh and flash-frozen/thawed Patient Sample 1. (**f**) Representative NAD(P)H τ_m_ and FAD τ_m_ images of organoids derived from fresh and flash frozen/thawed Patient Sample 2. Scale bar is 50 μm. (**g**) Cleaved caspase 3 staining, (**h**) Ki67 staining, (**i**) cell area, and (**j**) number of cells per organoid for organoids derived from fresh and flash-frozen/thawed Patient Sample 2. †p < 0.05 as indicated; *p < 0.05 versus Fresh. F = Fresh, N3/7 = Flash frozen and grown for 3/7 days. n = 5–10 organoids.

To test whether organoids could be grown from flash frozen primary human breast tumors, the method by which tumors are frequently preserved in human tissue banks, organoids derived from two primary human breast cancer biopsies were assessed. For Patient 1, organoids grew from both the fresh primary human tumor sample and from the flash-frozen/thawed sample (Fig. 5a, Supplementary Fig. 1). Immunofluorescence staining revealed similar percentages of cleaved caspase 3 positive and Ki67 positive cells between organoids generated from fresh tumor and organoids generated from flash-frozen tumor grown for 3 or 7 days (Fig. 5b,c). Likewise, there were no differences in the morphology of the cells, cell size, nucleus diameter or NCR ratio, between organoids generated from fresh tumor and organoids derived from the flash-frozen tumor (Fig. 5d, and Supplementary Fig. 2). However, there was a significant reduction in the number of cells per organoid in the organoids that were generated from the flash-frozen sample and grown for 3 days compared to organoids generated from fresh tumor from the same patient (Fig. 5e). No significant difference in number of cells within each organoid was detected for the organoids derived from flash-frozen tumor grown for 7 days versus organoids derived from fresh tumor from the same patient (Fig. 5e).

Likewise, organoids from Patient Sample 2 grew from fresh and flash-frozen tissues (Fig. 5f–h, Supplementary Fig. 1). No significant changes were observed in the percentage of cells that stained cleaved caspase positive or Ki67 positive between organoids derived from fresh tumor and organoids derived from flash-frozen tumor grown for 3 or 7 days (Fig. 5g,h). Likewise, no significant differences in cell morphology (cell area, nucleus diameter, or NCR) were detected between organoids derived from fresh tumor versus organoids derived from flash-frozen tumor for Patient 2 (Fig. 5i, and Supplementary Fig. 2). The number of cells within each organoid remained unchanged in organoids derived from flash-frozen tumor verses organoids derived from fresh tumor for Patient 2 (Fig. 5j).

### Drug Response of Organoids Generated from Flash-Frozen Human Breast Cancer Biopsies Resembles Drug Response of Organoids Generated from Fresh Tissue

**Figure 6:**
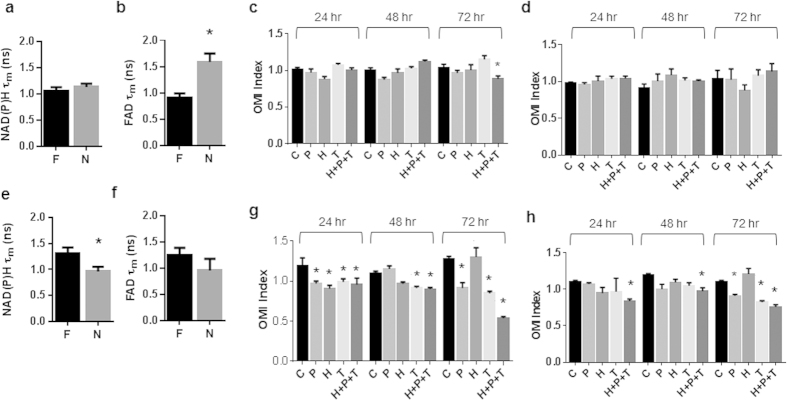
Drug response of organoids derived from flash-frozen thawed human breast tumors resembles drug response of organoids derived from fresh tissue. (**a**) NAD(P)H τ_m_ and (**b**) FAD τ_m_ measured from Patient Sample 1 organoids derived from fresh or flash-frozen tissues. *p < 0.05, versus Fresh. F = Fresh, N = flash frozen. Drug response of Patient Sample 1 organoids derived from fresh tissue (**c**) and flash-frozen tissue grown for 7 days (**d**) at 24, 48, and 72 hr. (**e**) NAD(P)H τ_m_ and (**f**) FAD τ_m_ measured from Patient Sample 2 organoids derived from fresh or flash-frozen tissues. *p < 0.05, versus Fresh. Drug response of Patient Sample 2 organoids derived from fresh tissue (**g**) and flash frozen tissue grown for 7 days (**h**) at 24, 48, and 72 hr. *p < 0.05 versus control; mean + /− SE. n = 30–300 cells.

As with the xenograft tumors, OMI measurements of drug response were compared between organoids derived from fresh and flash-frozen patient tumor samples. Both samples were derived from estrogen receptor positive, HER2 negative patients and the subsequent organoids were treated with the clinically relevant drugs, paclitaxel (P), trastuzumab (H), tamoxifen (T), and the combination, H + P + T. For Patient Sample 1, no difference in NAD(P)H τ_m_ was detected between the organoids generated from fresh tumor and flash-frozen tumor grown for 7 days (Fig. 6a). However, a significant increase in FAD τ_m_ was detected for organoids generated from flash-frozen versus fresh tumor from Patient 1 (p < 0.05, Fig. 6b). The OMI index was significantly decreased with H + P + T after 72 hr of treatment in organoids derived from fresh tumor from Patient 1 (Fig. 6c). None of the treatments induced a significant reduction in the OMI index for the organoids generated from flash-frozen tumor, grown for 3 (Table 1) or 7 days (Fig. 6d).

Finally, organoids derived from Patient Sample 2 showed significant response to the anti-cancer drugs. The NAD(P)H and FAD lifetime images and quantification (Figs 5a,f and 6a,b,e,f) indicate differing basal metabolic states of the organoids derived from fresh Patient Samples 1 and 2. First, NAD(P)H τ_m_ was significantly reduced in the organoids derived from the flash-frozen tumor compared to the organoids derived from fresh tumor (Fig. 6e), but the FAD τ_m_ showed no significant difference (Fig. 6f). The OMI index was significantly reduced in organoids derived from fresh tumor due to all drug treatments at 24 hr, tamoxifen and H + P + T at 48 hr, and paclitaxel, tamoxifen, and H + P + T at 72 hr (Fig. 6g). Likewise, the paclitaxel, tamoxifen, and H + P + T treated organoids derived from the flash-frozen tumor showed significant reductions in OMI index at 72 hr (Fig. 6h), while only the combination treatment, H + P + T, induced reductions in the OMI index at 24 and 48 hr. Altogether, these results suggest that viable organoids can be grown from frozen tumors and the cells remain sensitive to anti-cancer drugs.

### DMSO Frozen Tissue Yielded Organoids with Fewer Drug Response Inconsistencies than Flash Frozen Tissue

OMI measured drug response was compared between fresh-tissue generated organoids and organoids generated from frozen-thawed tissues. Both freezing methods, flash frozen and slow, DMSO frozen were compared for optimal drug response outcomes, as were the recovery times, 3 or 7 days, between organoid generation and drug treatment. The OMI index of the drug response significantly correlated between organoids derived from fresh and frozen tissues, for both freezing protocols (p < 0.0001, Supplementary Fig. 3). The response of organoids grown for 7 days and treated for 72 hr better reflected the response of fresh-tissue generated organoids with only 4/24 (16%) inconsistences versus 7/24 (30%) inconsistencies for organoids grown for 3 days before treatment (Table 1). The response of the organoids derived from DMSO frozen tissues resembled that of the organoids derived from fresh tissues after 72 hr of drug treatment with fewer inconsistencies than flash-frozen preparations, 3/16 (19%) vs. 9/32 (28%) (Table 1). The inconsistency rate for OMI measured drug response of organoids grown derived from DMSO frozen tissues and grown for 7 days prior to drug treatment was 1/8 (12.5%).

## Discussion

Primary cells grown in 3D culture as organoids provide a robust and relevant model for investigations of cancer progression, drug development, and individualized treatment planning in the clinic. However, current organoid generation protocols require fresh tissue^9^,^11^, which limits the use of primary human tumor organoids in research studies and clinical applications. This study evaluated the viability and OMI-measured drug response of both xenograft and primary human organoids generated from tissue preserved by two methods of freezing:, (1) flash-frozen in liquid nitrogen, or (2) slowly frozen in media supplemented with 5% DMSO. Flash freezing is the current method for tissue banking yet slow freezing in DMSO is known to preserve tissue viability^12^. For all samples, organoids were generated from a piece of fresh tissue and compared with organoids generated from a portion of the same tumor that was frozen and thawed, thus organoids derived from fresh and frozen tissues were from the same donor to eliminate donor variability and ensure all significant findings are due to the experimental freezing protocols. While OMI has been performed on organoids to evaluate drug response^9^, this is the first study to grow organoids from frozen tissues and the first study to evaluate the basal and drug treated metabolism of organoids derived from frozen tissues.

The success rate of organoid generation was 100% for both the xenograft tissues (Fig. 1) and the human biopsies (Fig. 5); all fresh (4/4 samples), flash-frozen thawed (4/4 samples), and DMSO-frozen-thawed (2/2 samples) tissue samples. While only observed in 1 sample (HR6), the changes in cell morphology (Fig. 2e,f) agree with prior studies of frozen/thawed cells that have demonstrated reduced cell volumes following flash freezing, and these morphological differences are reduced in slow-frozen cells^12^.

The OMI index is a robust, dynamic endpoint of cellular metabolism and has been shown to accurately measure early drug response in anti-cancer drug-treated cells, tumors imaged *in vivo*, and organoids^9^,^13^,^14^. OMI-measured drug response correlates with and accurately matches *in vivo* drug response^9^. The OMI index of drug-treated organoids was significantly correlated between fresh and frozen preparations for all tissue samples (Supplementary Fig. 3). Drug induced increases in apoptosis and decreases in proliferation verify that a decrease in the OMI index of tumor organoids correlates with drug response (Figs 3 and 4), ensuring the OMI index reports drug response in organoids derived from frozen tissues. Furthermore, the drug resistant samples, HR6 and Patient Sample 1, maintained drug resistance in the organoids derived from frozen-thawed tissue, confirming treatment behavior of organoids derived from frozen samples. However, the frozen/thawed tissues were less sensitive to anti-cancer drugs, as evidenced by a dampened dynamic range of the OMI index (Fig. 3c–e, 4c–e and 6g,h) and fewer drug-induced responses (Table 1). The increased number of discrepancies of drug response for organoids derived from flash frozen tissue (28.125% vs. 18.75% for DMSO frozen tissues) indicate the flash freezing protocol may select for more resistant cells. Increased primary tumor proliferation rate (Patient Sample 1) was observed with increased drug resistance of organoids derived from fresh and frozen organoids (Fig. 6); however, the increased proliferation did not increase cell survival of the freezing process (Fig. 5). The mutations and phenotypic differences that enable cells to evade drug treatment may enable the cells to survive the flash-frozen protocol.

The dynamic range of the drug-induced metabolism changes may be reduced due to damage caused by the freezing protocols on metabolic enzymes and substrates^15^. Indeed, the significant changes in NAD(P)H and FAD fluorescence lifetimes observed in all four tissue samples (Figs 3a, 4b and 6b,e) indicate that the freeze/thaw protocols induce significant biochemical changes within the cells. The changes in the free lifetime components (τ_1_ for NAD(P)H and τ_2_ for FAD, Supplementary Fig. 4) suggest post-freezing morphological changes to NAD(P)H and FAD, and changes in the microenvironment surrounding NAD(P)H and FAD^16^,^17^,^18^. The changes in protein-bound fluorescence lifetimes (τ_2_ for NAD(P)H and τ_1_ for FAD, Supplementary Fig. 4) may be indicative of morphological changes in the enzyme-substrate conformations, changes in preferred protein binding, or differences in the microenvironment of surrounding NAD(P)H and FAD enzyme structures^16^,^17^. Changes in the relative weights of the free and protein-bound lifetimes suggest an increase in the proportion of free NAD(P)H and FAD relative to protein-bound forms (a_1_ for NAD(P)H and FAD, Supplementary Fig. 4). A previous study of *in vivo*, freshly excised tissue, and frozen hamster cheek pouch tissues also identified an increased FAD τ_m_ in frozen tissues^19^. A cryopreservation study has previously identified freezing-induced inactivation of metabolic enzymes, such as lactate dehydrogenase^15^, indicating changes in metabolism coenzyme environment after tissue freezing. The freezing process may introduce additional changes to cellular proteins, oncogenes, and signaling. Future experiments are warranted to investigate these effects of freezing.

In order to investigate whether cells required a recovery growth period following freeze/thaw protocols, all experiments were repeated on organoids grown for 3 or 7 days. These results (Figs 1,2,5, and Supplementary fig. 5) suggest that the biochemical and morphological effects of the freezing process persist for at least a week of growth. However, additional OMI drug response (12.5% fewer inconsistencies) was detected in organoids grown for 7 days (Table 1) versus 3 days, suggesting a longer recovery time yields response data more consistent with that of fresh organoids.

Traditional organoid generation protocols require immediate processing of fresh tissue into organoids, which limits the application of organoid-based experiments and drug-response studies to medical centers with both operating rooms and research facilities. The results of this study demonstrate that organoids can be grown and are viable when generated from flash-frozen and DMSO-frozen tissues. The OMI-measured drug response of organoids derived from frozen tissues correlates with that of organoids derived from fresh tissue; however, freezing induced cellular changes reduced the drug response of organoids derived from once-frozen tissues. While not directly tested in primary human tissue samples due to limited primary tissue, triplicate experiments in primary xenograft organoids indicate organoids derived from DMSO-frozen bulk tissue most reflect the morphology, metabolism coenzyme environment, and drug response of fresh tissues. Therefore, when possible, bulk tissue should be slowly frozen in DMSO-supplemented media for preservation and later organoid generation. However, organoids may be grown and used for drug response analysis from flash-frozen tissues when DMSO is not available or for tissue samples already flash-frozen, such as in human tissue banks, as long as the researcher is aware that the freezing process may introduce cellular differences and reduced drug response.

## Methods

### Mouse Xenografts

This study was approved by the Vanderbilt University Animal Care and Use Committee and meets the NIH guidelines for animal welfare. BT474 or HR6 cells (10^8^) in 100 μl Matrigel were injected in the inguinal mammary fad pads of female athymic mice (J:NU; The Jackson Laboratory). BT474 and HR6 tumors are both estrogen receptor positive and HER2 overexpressing. The HR6 tumors were extracted from a BT474 xenograft that developed acquired resistance to the HER2 antibody trastuzumab *in vivo*^20^. The HR6 cells and tumors used herein retain HER2 overexpression^9^,^20^, thus the BT474 and HR6 tumors represent trastuzumab responsive and trastuzumab-resistant tumors, respectively. In this study, one BT474 tumor and one HR6 tumor were used. When tumors were ~500 mm^3^, mice were humanly sacrificed and the tumors removed. Each tumor was cut into three approximately equal sections of ~150 mm^3^. One section was processed immediately into organoids, one section was flash frozen, and one section was slowly frozen in media supplemented with 5% DMSO. The matched samples reduced donor variability to ensure all differences in experimental groups are due to freezing methods.

### Clinical Breast Cancer Samples

This study was approved by the Vanderbilt University Institutional Review Board and informed consent was obtained from all subjects. Two primary tumor biopsies from separate patients were provided by an expert breast pathologist (ME Sanders). Both tumors were from high-grade primary tumors, expressed estrogen receptors and progesterone receptors and lacked overexpression/amplification of HER2. Patient Sample 1 had a high rate of proliferation while Patient Sample 2 had an intermediate rate of proliferation. Tumor biopsies were obtained rapidly *ex-vivo* from the surgical excision specimen. The tumor biopsies were placed in sterile DMEM and transported on ice to the laboratory (~5 minute walk). Previous studies indicate that this tissue handling protocol maintains tissue viability and OMI endpoints^9^,^19^. The tumor biopsies were cut into two approximately equal sections, ~200 mm^2^. One section was processed into organoids immediately, and the second section was flash frozen.

### Freezing Protocols

For flash freezing in liquid nitrogen, samples were placed in a histology cassette and submerged in liquid nitrogen for 30 seconds. Then, the cassette was removed, wrapped in foil, and stored in a −80 °C freezer. For the slow freezing method, samples were placed in a cryotube with 950 μl tissue culture media and 50 μl DMSO. Samples were slowly frozen by placement in a Styrofoam container in a −80 °C freezer. Samples were stored at −80 °C until use, 6–12 months.

### Organoid Generation

Frozen samples were thawed in PBS at room temperature for ten minutes. All samples were washed three times with sterile PBS. Tissue samples were placed in 0.5 ml primary mammary epithelial cell (PMEC) media [DMEM:F12 + EGF (10 ng/ml) + hydrocortisone (5 μg/ml) + insulin (5 μg/ml) + 1% penicillin:streptomycin] and dissociated into macrosuspensions of tissue approximately 50–300 μm in diameter by mechanical cutting of the tissue with a scalpel and surgical scissors. Matrigel was added to the macrosuspension solutions in a 2:1 ratio and 100 μl of the matrigel/macrosuspension solution was placed on coverslips. The gels solidified at room temperature for 30 minutes and then in the incubator for 1 hour. Gels were overlaid with PMEC media.

Organoids generated from fresh tissues were treated immediately. BT474 and HR6 organoids were treated with the following drugs and combination: control (human control IgG + DMSO), trastuzumab (25 μg/ml), paclitaxel (25 nmol/L), the PI3K inhibitor XL147 (25 nmol/ml), and trastuzumab + paclitaxel + XL147. The BT474 and HR6 organoids were treated with clinically used drugs, paclitaxel (chemotherapy) and trastuzumab (anti-HER2 antibody), to evaluate clinically used drugs. Additionally, we treated the BT474 and HR6 organoids with an experimental drug, XL147 which is a PI3K inhibitor in clinical trials to overcome trastuzumab resistance of HER2-overexpressing tumors^21^,^22^. The organoids derived from the clinical biopsies were treated with control (human control IgG + DMSO), trastuzumab (25 μg/ml), paclitaxel (25 nmol/L), tamoxifen (2 μmol/ml), and trastuzumab + paclitaxel + tamoxifen. The receptor expression of the organoids derived from clinical biopsies was unknown at the time of sample acquisition and treatment. Therefore, these organoids were treated with three clinically used drugs, a chemotherapy (paclitaxel), a ER antagonist (tamoxifen), and a HER2 antibody (trastuzumab). The combination treatment group assessed whether drugs have added benefit when used in combination. Organoids derived from frozen tissues were grown for a recovery period of 3 or 7 days (media replaced every 2–3 days) and then treated with the same drugs and combination as the fresh-tissue derived organoids. Each sample received only one drug treatment.

### Optical Metabolic Imaging

Fluorescence lifetime imaging was performed on a multiphoton microscope (Bruker) modified for fluorescence lifetime imaging, as previously described^9^,^13^,^19^. Briefly, a titanium-sapphire laser, tuned to 750 nm for NAD(P)H excitation and tuned to 890 nm for FAD excitation, provided the excitation light. A 40X objective (1.3 NA) coupled excitation and emission light. Customized filter sets isolated NAD(P)H emission between 400–480 nm and FAD emission between 500–600 nm. A GaAS PMT (H7422P-40; Hamamatsu) detected emitted photons and time correlated single photon counting electronics (SPC-150; Becker and Hickl) enabled fluorescence lifetime imaging. Images, 256 × 256 pixels, were collected for 60 s with a pixel dwell time of 4.8 μs. Photon count rates were maintained above 5 × 10^5^ to ensure adequate photon counts for lifetime fits and no photobleaching occurred. The instrument response full width at half maximum was 260 picoseconds as measured from the second harmonic generation of a urea crystal. Daily fluorescence lifetime validation was confirmed by imaging a fluorescent bead (Polysciences Inc.). The measured lifetime of the bead (2.1 ± 0.04 nanoseconds) agrees with published values^9^,^13^,^23^,^24^.

NAD(P)H and FAD fluorescence lifetime images of the organoids were acquired at 24, 48, and 72 hours after drug treatment. For each organoid, the NAD(P)H image was acquired first and followed immediately by the FAD image. Organoids were imaged through glass coverslips on the inverted microscope. One image from each of six representative organoids were collected per group (6 organoids/group; n = 30–300 cells/group), at an imaging depth through the center of the organoid.

Fluorescence lifetime images were analyzed as described previously^9^,^13^,^25^. Briefly, fluorescence lifetime decay curves were deconvolved from the measured instrument response function and fit to a two component model, 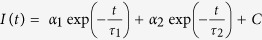, where I(t) is the fluorescence intensity at time *t* after the laser pulse, α_1_ and α_2_ are the fractional contributions of the short and long lifetime components, (i.e. α_1_ + α_2_ = 1), τ_1_ and τ_2_ are the fluorescence lifetimes of the short and long lifetime components, and C accounts for background light (SPCImage). A two component model was used to fit the lifetime decays of NAD(P)H and FAD because NAD(P)H and FAD exist physiologically in both free and protein-bound forms^17^,^18^. The free and protein-bound forms have different lifetimes depending on fluorescence quenching^16^. NAD(P)H self-quenches in the free state and thus has a short lifetime associated with free NAD(P)H and a long lifetime with bound NAD(P)H^16^,^17^. FAD is quenched when bound and has a short bound lifetime and long free lifetime^18^. The mean lifetime, τ_m_, is calculated as a weighted average of τ_1_ and τ_2_, τ_m_ = α_1_ * τ_1_ + α_2_ * τ_2_.

Using an automated cell segmentation routine in CellProfiler, the images were segmented into cells, nuclei, and cytoplasms^25^. The following endpoints were extracted from the fluorescence lifetime data sets for both the NAD(P)H and FAD image for each cell: fluorescence intensity, mean fluorescence lifetime, α_1_, τ_1_, and τ_2_. A redox ratio image was computed by dividing the NAD(P)H intensity by the FAD intensity at every pixel in the image. The average redox ratio for each cell was also extracted. As determined previously, the optical redox ratio, NAD(P)H mean lifetime, and FAD mean lifetime are independent measures of cellular metabolism^13^ and a combination index, the optical metabolic imaging index, provides a robust endpoint for evaluating drug response^9^. The OMI index was calculated for each cell as follows^9^,


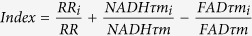


### Immunofluorescence

Immunofluorescence labeling of cleaved caspase 3 and Ki67 was performed as previously described^9^,^26^. Briefly, gels were fixed with 2 mL of a 4% paraformaldehyde solution and neutralized with a 0.15 mol/L glycine solution. A 0.02% Triton X-100 solution was used to permeabolize cellular membranes. Gels soaked in a blocking solution (1% fatty acid-free BSA, 1% donkey serum) over night at room temperature. The next day, gels were incubated for 30 minutes at room temperature with 100 μl of the primary antibody solution: either anti-cleaved caspase 3 (Life Technologies) or anti-Ki67 (Life Technologies) diluted 1:100 in a 1% donkey serum PBS solution. Gels were washed 3X with PBS and 100 μl of the secondary antibody solution (goat anti-rabbit IgG FITC probe diluted 1:100 in a 1% donkey serum PBS solution) was added to each gel. Gels were washed 3X with PBS and 2X in water then mounted on slides with 30 μl of ProLong Antifade Solution (Molecular Probes). Positive staining was confirmed in mouse small intestine for Ki67 and mouse thymus for cleaved caspase 3. Immunofluorescence results are presented as the percentage of positively stained cells from 6 organoids imaged at 40X (1.3 NA). Representative immunofluorescence images are presented in Supplementary Figure 6.

### Quantification of Cell Morphology

Cellular morphology was assessed from the NAD(P)H fluorescence lifetime image, integrated over time to yield an intensity image. Average cell size and nuclear-to-cytoplasm ratio (NCR) were extracted from the CellProfiler segmentation routine outputs. Average nucleus diameter was determined by manually measuring the longest axis through the center of each cell in ImageJ.

### Statistical Analysis

A non-parametric, Kruskal-Wallis 1 way ANOVA test was used to test for significant differences within immunofluorescence and morphology results (n = 5–12 organoids). Dunn’s multiple comparisons test was sequentially preformed following a significant ANOVA finding to identify groups with significantly different mean values. FLIM control data was assessed for normality using the D’Agostino & Person Omnibus Normality Test (n = 30–300 cells). All FLIM data was assessed with parametric statistical tests because 9/12 control FLIM data sets passed the normality test (p > 0.05) and the large sample size (>30) minimizes error due to normality assumption. A student’s t-test with a Bonferoni correction for multiple comparisons was used to assess differences in fluorescence lifetime endpoints between fresh and frozen control organoids. Time course drug response OMI data was analyzed with 2-way ANOVA followed by Tukey’s Multiple Comparisons test (n = 30–300 cells). A nonparametric Spearman’s Correlation test was used to evaluate the correlation between OMI index values for drug-treated organoids derived from fresh or frozen tissue samples. For all statistical comparisons, an alpha level of 0.05 was used for significance.

## Additional Information

**How to cite this article**: Walsh, A. J. *et al.* Drug response in organoids generated from frozen primary tumor tissues. *Sci. Rep.* **6**, 18889; doi: 10.1038/srep18889 (2016).

## Supplementary Material

Supplementary Information

## Figures and Tables

**Table 1:**
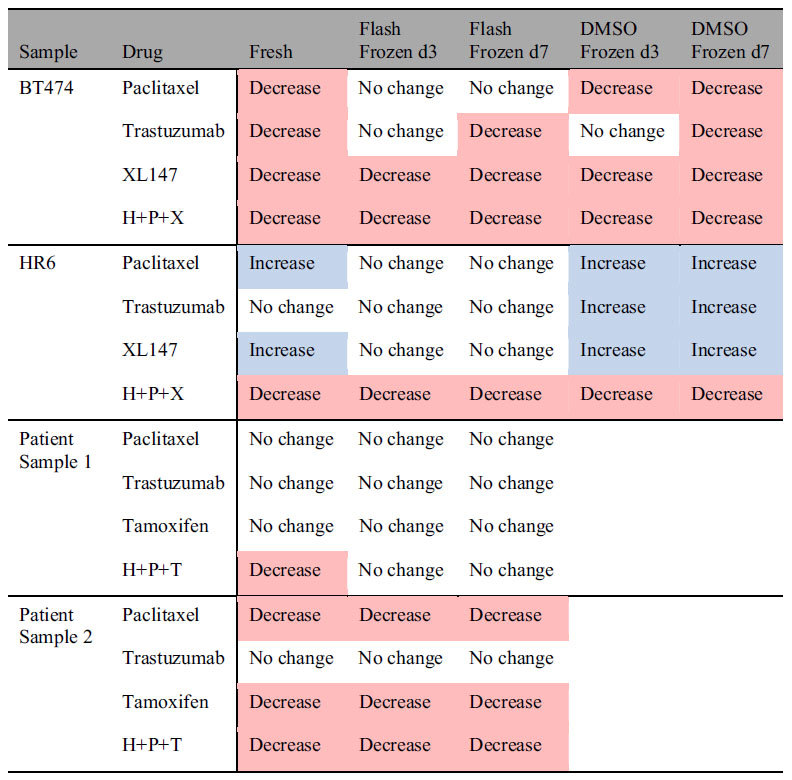
Drug induced changes in OMI index at 72 hr drug treatment for different organoid generation protocols.
