# Author Correction: Drug response in organoids generated from frozen primary tumor tissues

**DOI:** 10.1038/s41598-019-42133-w

**Published:** 2019-04-24

**Authors:** Alex J. Walsh, Rebecca S. Cook, Melinda E. Sanders, Carlos L. Arteaga, Melissa C. Skala

**Affiliations:** 1 Department of Biomedical Engineering, Vanderbilt University, Station B, Box 1631, Nashville, Tennessee 37235 USA; 2 Department of Cancer Biology, Vanderbilt University, 2220 Pierce Avenue, Nashville, Tennessee USA; 3 Department of Medicine, Vanderbilt University, 2220 Pierce Avenue, Nashville, Tennessee USA; 4 Breast Cancer Research Program, Vanderbilt- Ingram Cancer Center, Vanderbilt University, Nashville, USA; 5 Department of Pathology, Microbiology & Immunology, Vanderbilt University Medical Center, 1301 Medical Center Drive 4918-A TVC Building, Nashville, Tennessee 37232 USA

Correction to: *Scientific Reports* 10.1038/srep18889, published online 07 January 2016

The Competing Interests section in this Article is incorrect and should read:

“Carlos Arteaga served as a consultant for Genentech, the manufacturer of one of the drugs evaluated in the paper. The other authors declare no competing financial interests.”

