# Supplementary material for: Drug response in organoids generated from frozen primary tumor tissues
Source: Sci Rep. 2016 Jan 7;6:18889. doi: 10.1038/srep18889 (PMC4703961; doi:10.1038/srep18889)
Supplement: Supplementary Information [file srep18889-s1.pdf]

Supplementary Information for

"Drug response in organoids generated from frozen primary tumor tissues"

Alex J. Walsh, Rebecca S. Cook, Melinda E. Sanders, Carlos L. Arteaga, Melissa C. Skala

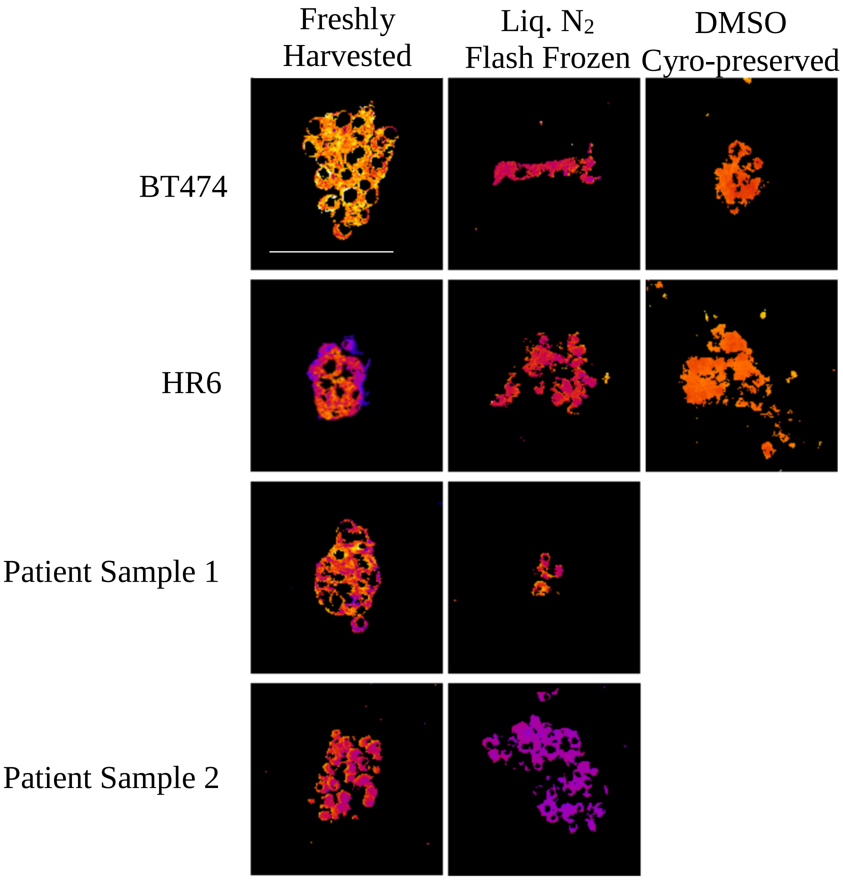

Supplementary Fig. 1: Representative redox ratio images of BT474, HR6, Patient Sample 1, and Patient Sample 2 organoids derived from fresh, flash frozen, and DMSO frozen tissues. Scale bar is 100 μm.

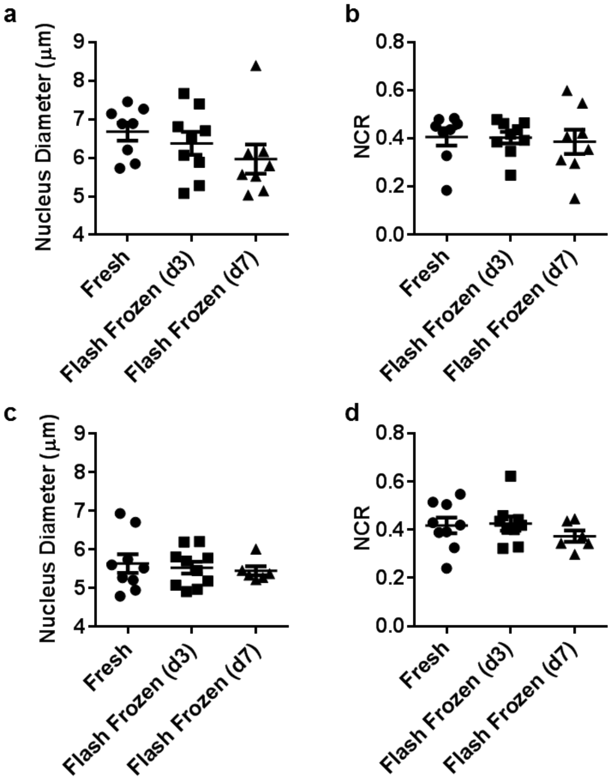

Supplementary Fig. 2: (a) Nucleus diameter and (b) nuclear to cytoplasm ratio (NCR) for organoids derived from fresh and flash-frozen tissues of Patient Sample 1. (c) Nucleus diameter and (d) nuclear to cytoplasm ratio (NCR) for organoids derived from fresh and flash-frozen tissues of Patient Sample 2. d3/d7 represents organoids grown for 3 or 7 days after generation. n = 5-10 organoids.

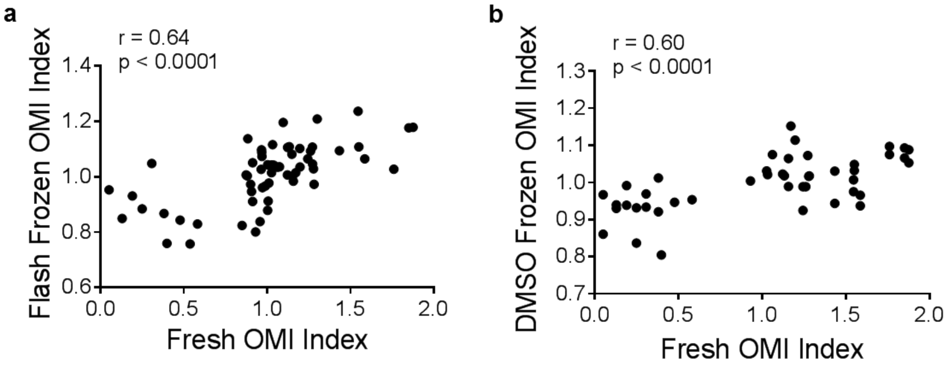

Supplementary Fig. 3: Correlation between OMI index values for drug response studies of organoids derived from fresh and flash-frozen thawed tissue (a) or DMSO frozen tissue (b). r is Spearman's correlation coefficient.

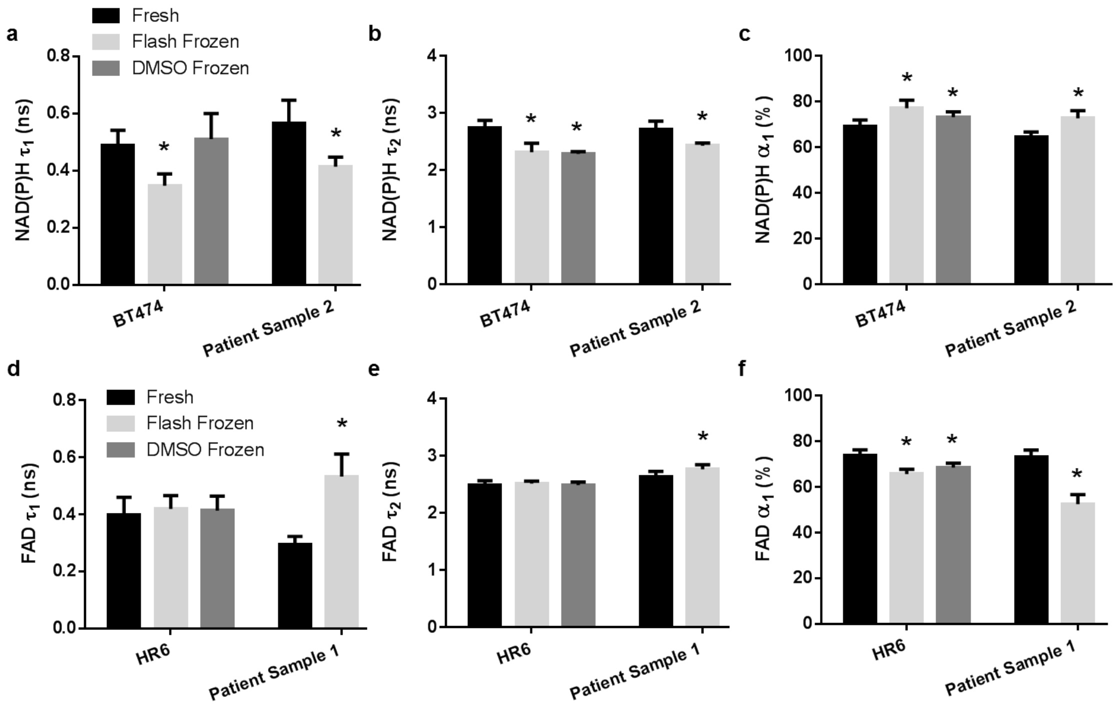

Supplementary Fig. 4: (a) NAD(P)H τ_1, (b) NAD(P)H τ_2, and (c) NAD(P)H α_1 for organoids derived from fresh tissues and organoids derived from frozen tissues and grown for 7 days. (d) FAD τ_1, (e) FAD τ_2, and (f) FAD α_1 for organoids derived from fresh tissues and organoids derived from frozen tissues and grown for 7 days. * $p<0.05$ vs. Fresh; mean +/- SE. n = 30-300 cells.

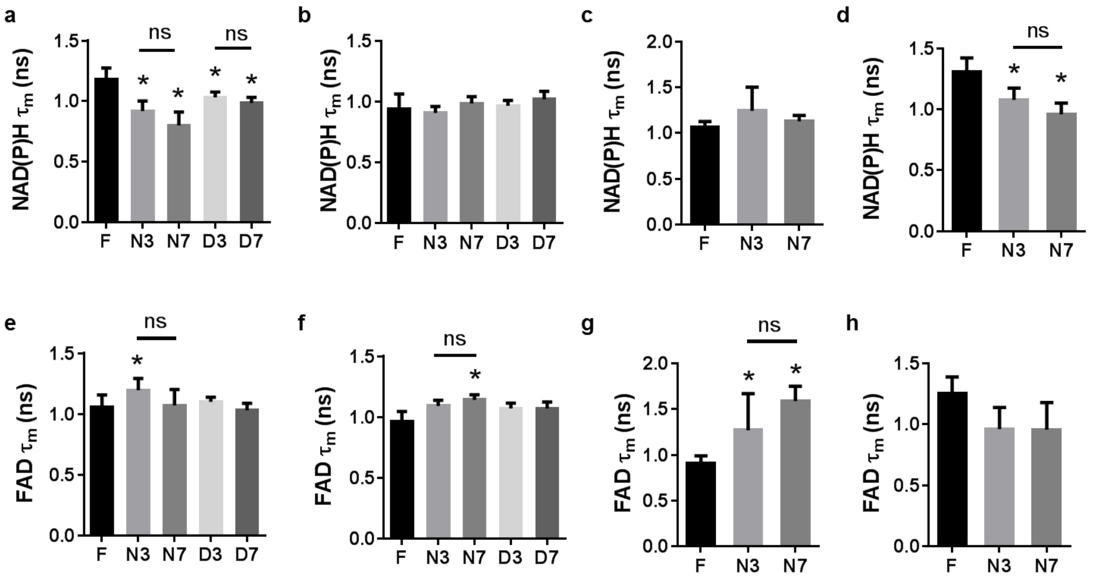

Supplementary Fig. 5: NAD(P)H τ_m of (a) BT474, (b) HR6, (c) Patient Sample 1, and (d) Patient Sample 2 organoids grown from fresh and frozen tissue. FAD τ_m of (e) BT474, (f) HR6, (g) Patient Sample 1, and (h) Patient Sample 2 organoids grown from fresh and frozen tissue. d3/d7 represents organoids grown for 3 or 7 days after generation. F = Fresh, N3/7 = Flash frozen and grown for 3/7 days, D3/7 = DMSO frozen and grown for 3/7 days. * $p<0.05$ vs. Fresh; ns = not significant, mean +/- SE. n = 30-300 cells.

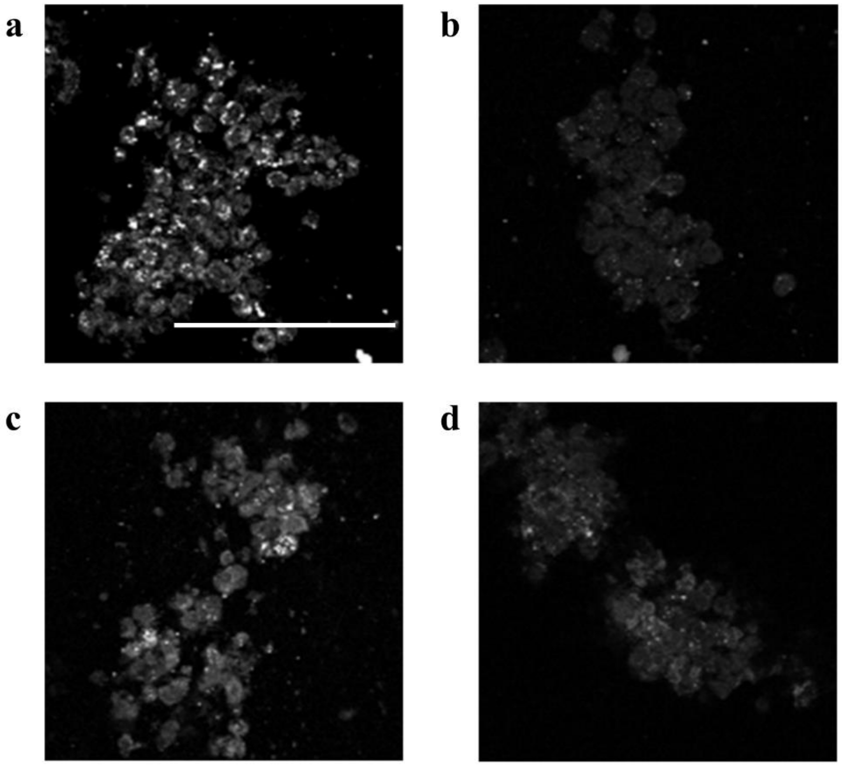

Supplementary Fig. 6: Representative immunofluorescence images of HR6 organoids derived from fresh tissue (a,b) or flash frozen tissue (c,d) and stained for Ki67 (a,c) and cleaved caspase 3 (b,d). Scale bar is 100 μm.
